# Artificial Production of Variations in Types

**Published:** 1892-06

**Authors:** C. O. Whitman


### Artificial Production of Variation in Types.

In reply to your request for a few words on the question of artificial production of variations, as presented by Mr. West in *Science* of April 22. I may say that I quite agree with Mr. West in thinking that all attempts to produce new species by mutilations of the parents are foredoomed to failure. The idea that the embryo is in any sense a *reflected* image of the parent, and consequently that any particular loss or modification of an organ in the parent during the adult life must impress itself upon the embryo, has not a shadow of a basis in embryology.

Mr. West asks: "Would it not seem the proper and only method to study the laws governing the modifications of the embryo?" If we substitute germ-cells for "embryo," the question may be answered affirmatively. If the question, as it stands, implies that modifications received during *embryonic* life, as the result of external influences, would be any more likely to repeat themselves in the next generation than if acquired during adult life, I should say that the assumption is entirely unwarranted.

The form and features of the adult are predetermined in the constitution of the germ-cell. No one denies that external conditions and influences may affect more or less the course of development; but the specific form of the adult is already settled in the germ before development begins. These are mere truisms in embryology. C. O. WHITMAN.

